# Mass Yields, Antioxidant and Anti-DU145 Prostate Cancer Cell Proliferation Properties of ProSoy Soymilk as Affected by Extraction Methods and Cooking

**DOI:** 10.3390/antiox13070755

**Published:** 2024-06-21

**Authors:** Sam K. C. Chang, Yingying Tan

**Affiliations:** 1 Department of Food Science, Nutrition and Health Promotion, Mississippi State University, Mississippi State, MS 39762, USA; 2 Experimental Seafood Processing Laboratory, Coastal Research and Extension Center, Biloxi, MS 39567, USA; 3 Department of Chinese Medicine, Shaanxi University of Chinese Medicine, Xianyang 712046, China; tanyy@sntcm.edu.cn

**Keywords:** ProSoy yellow soybean, soymilk processing methods, phenolics and isoflavones, antioxidant capacity, anti-DU145 prostate cancer proliferation

## Abstract

Both the soybean variety and processing method affect the end soybean product’s characteristics. This study’s objective was to characterize the effects of four extraction methods (variations of soaking and grinding) combined with cooking on the content and composition of phenolic substances and the antioxidant and anti-DU145 prostate cancer cell proliferation properties of soymilks prepared from a yellow soybean of the ProSoy variety, which is a high-protein variety. The results showed that the soymilk processing yield was the greatest using method 4, although method 2 gave the highest solid and protein yields by about 14 and 12%, respectively. Method 4, a two-step grinding method, also gave increased yields (8 and 7% for solids and proteins, respectively), and in all but one instance produced higher total phenolic content (TPC), total flavonoid content (TFC), condensed tannin content (CTC), and total isoflavone content values in both raw and cooked soymilks as compared to method 1. Cooking the soymilks reduced 14–17% of their total phenolic substances. Cooking reduced the anti-cancer capacity of the phenolic extracts from the soymilk prepared using method 4 by increasing the IC50 value from about 4.9 mg/mL to 6.8 mg/mL. The increases in phenolic compounds and antioxidants produced in the Prosoy soymilks using methods 2 and 4, with simultaneous increases in product and solid yields, are of significant benefit to the soymilk industry and consumer health.

## 1. Introduction

Soymilk is a traditional non-dairy beverage that has been consumed in the East Asian countries for thousands of years [1], which is gaining popularity in the Western world due to the fact that soy has health benefits in the prevention of chronic diseases, possibly related to the presence of isoflavones and phenolic antioxidant substances [2,3,4,5,6]. In addition, soy foods (non-ultra-processed formulated foods) provide nutritious plant proteins that are free of cholesterol and are heart-healthy, with an FDA-approved heart health claim [7,8], making them excellent choices for vegetarians. Soymilk contains no lactose and is a good choice for people who are intolerant to lactose and dairy proteins. Soybeans that are commercially grown have special nutritional quality characteristics [9]. Poysa and Woodrow [10] reported that the yield/kg seed dry matter and solid content values of soymilk and tofu were significantly affected by the genotype of soybean. There are basically two types of soybeans according to the purpose of usage. Some varieties are genetically bred for high oil contents, particularly high oleic fatty acid contents, to produce soy oil, while the defatted soy meal is bred for use as animal feed. The other type is high-quality food-grade soybean, which is bred for making foods, such as soymilk, tofu, bean sprouts, natto, and tempeh. Soymilk is also used as an intermediate product for tofu manufacturing [11], during which a coagulant is added to form tofu gels. High protein contents are favorable for human nutrition and for gelation in tofu making. In the soymilk beverage industry, soybean genotypes with high yields of soymilk, along with high solid and protein contents, are highly desired and are the targets in breeding for food and nutritional quality enhancements. The soybeans used for making soymilk and tofu need to be soaked in water prior to grinding to extract the soluble nutrients into the soymilk. Therefore, easily hydrated seeds are preferred, since soaking beans well prior to grinding leads to higher product and solid yields. Black soymilk is made from specialty soybeans that have long been considered a source of food and medicine in the Chinese *Ben Cao Gang Mu*—the Compendium of Materia Medica [12]. Black soymilk is traditionally regarded as a health-promoting beverage and is more expensive than yellow soymilk. However, the vast majority of commercial soymilk is made from yellow soybeans, since their planting yield is higher than for black soybean; therefore, the price of yellow soybean is generally 2–3 times lower than for black soybean. In addition to soaking, wet grinding is a critical step in the soymilk extraction process that can significantly affect the product yield and retention of the bioactive components.

Cancer is the second leading cause of death in the United States, exceeded only by heart disease. Prostate cancer is the second most common cancer in men in the United States, and is the third cause of male cancer deaths worldwide. In the USA, prostate cancer ranks first among new cases of all cancers in men. In 2020, the latest available incidence data showed that 201,082 new cases of prostate cancer were reported among men, while 32,707 men died of the same cancer, representing 19% of the death rate [13]. Epidemiological studies have shown that the prostate cancer mortality rate is commonly lower in East Asian males than in Western males, which is believed to be partly due to the frequent consumption of soy products in their diet [14].

The isoflavones in soy have been demonstrated to contribute to reductions in prostate cancer cells in ovariectomized mice [15,16,17,18]. Studies [19,20,21] suggest that the degree of processing negatively affects the bioactivities of soy products. Furthermore, Hsu and others [22] found that whole soybean flour extract (similar to raw soymilk) was more potent and safer than individual isoflavones or their combinations for inhibiting prostate cancer growth. The recent research by Yu et al. [23] compared the effects of several processing methods (oven drying, wet soaking without drying, and boiling the extracted slurry or not prior to filtration) on the phenolics and antioxidant activities of soybean products made from 15 soybean breeds in China. The study concluded that wet soaking the soybeans without prior oven drying produced better antioxidant profiles. However, the cooking conditions of 5 min at 100 °C meant they retained 30% of the trypsin inhibitors in the soybeans, which would not be desirable for optimizing the protein content and nutritional value [24]. In addition, these above-mentioned studies related to soymilk processing and our recent research [25] using hot grinding to eliminate trypsin inhibitors and the ‘beany’ flavor did not consider the soymilk yields, solid contents, and protein recovery rates, which are important to the economy of the food industry.

Our previous publication [26] characterized the compositions of the phenolic extracts, including isoflavones, from black soybean soymilk and okara, as well as their potential effect on the inhibition of prostate cancer cells in vitro. The selection of black soybean was based on our previous study, which showed that black soybean possessed significantly higher phenolic substance contents and antioxidant activity rates than yellow soybean [27]. However, we discovered in a subsequent study [28] that the soymilks (either raw or cooked) from two yellow soybean varieties (Proto and IA2032) had higher oxygen radical absorbance capacity (ORAC) rates than that of the soymilk from black soybean, despite having lower total phenolic contents. The results indicate that yellow soybean also has potential to be a healthy beverage that is as good as black soymilk, which deserves further investigation.

Generally, four processing steps are involved in making soymilk: (1) soaking the soybeans; (2) grinding the soybeans in water; (3) filtering the soybeans to remove insoluble residues (okara); (4) heating the soybeans to destroy anti-nutrients and inactivate beany-odor-producing enzymes. Variations in the processing conditions of each of these steps may affect the soymilk quality. We have reported how four specific preparation and cooking methods could affect the bioactive components and their potential health effects in black soymilk and proved that a two-step grinding method could produce soymilk with higher antioxidant and anti-prostate cancer cell (DU145) property levels [26].

Our earlier research studies showed that the heating method affected the phenolic and antioxidant capacity levels of soymilks made using three different soybean varieties and four heating practices [28]. The results showed that a soymilk made from black soybean with green cotyledons had a higher total phenolic content and a higher free benzoic acid content than those made from Proto yellow soybean and the soybean variety IA-2032 released by Iowa State University [28]. Cooking reduced the total phenolic content and increased the total flavonoid content in the soymilks, and the extent of the reduction or increase were variety-dependent. However, the antioxidant capacity profiles indicated that the DPPH (2,2-diphenyl-1-picrylhydrazyl) free radical scavenging activity (referred to as DPPH assay) and FRAP (ferric-reducing antioxidant power) of the black soymilk were higher than the yellow soymilk, although the ORAC (oxygen radical absorbance capacity) was lower than that of the yellow soymilk. Poysa and Woodrow [10] found that the genotype is highly related to the soymilk yield and solid content. These findings showed that further research is needed to understand the behaviors of different varieties. ProSoy soybean is a food-grade, high-protein soybean variety with a good planting yield that was released by our team at the North Dakota State University for making soymilk and tofu [29]. Its antioxidant capacity and anti-prostate cancer potential as affected by the soymilk manufacturing processes have not been characterized. The soymilk product, solid, and protein yields are very important to the soymilk industry, since higher-yield varieties require less materials and offer reduced costs. On the other hand, a soymilk variety that produces a higher antioxidant level and disease prevention potential would benefit consumers more. Although the antioxidant properties of soy phenolics have been reported [9,11,20,30,31], the effects of various grinding methods, especially when in conjunction with the product and solid yields and cooking method, on the phenolic composition and antioxidant profiles have only been characterized for black soybean in our laboratory. This kind of research has never been reported for yellow soybean.

The objective of this study was to identify which of the four different combinations of cold soaking and grinding methods, as we designed previously [26], could be used to improve the product yields, solid contents, and protein yields, and simultaneously to improve the phenolic substance compositions, in vitro antioxidant profiles, and in vitro anti-prostate cancer DU145 cell proliferation properties of the soymilk made from ProSoy yellow soybean.

## 2. Materials and Methods

### 2.1. Soybean Materials and Chemicals

Dry matured soybean (*Glycine max* (L.) *Merr.*) of the ProSoy variety, a high-protein food-grade soybean (45.7% protein and 22.3% lipids) with a light-yellow seed coat, was obtained from Sinner Brothers and Bresnahan (Casselton, ND, USA). All analytical chemicals and phenolic and antioxidant standards were either obtained from VWR International (West Chester, PA) and Sigma-Aldrich Chemical Company (St. Louis, MO, USA) or purchased from Wako Chemicals USA (Richmond, VA, USA), as per the reports by Xu and Chang [9,28] and Tan et al. [26].

The human prostate cancer cell line DU145 was purchased from the American Type Culture Collection (ATCC, Manassas, VA, USA). The other chemicals associated with the cell culture experiments were either obtained from Cambrex Bio Science Walkersville, Inc. (Walkersville, MD, USA) or Mediatech, Inc. (Herndon, VA, USA), as described in our previously reported article [26].

### 2.2. General Procedures for Preparing Soymilk

General stepwise procedures for manufacturing bench-scale soymilk were carried out, according to the paper by Yuan and Chang [24]: (1) rinsing the soybeans thoroughly and soaking them in water at 4 °C overnight; (2) grinding the soaked soybeans; (3) separating the ground slurry into soymilk and okara (insoluble soy residue) using a muslin cloth; (4) cooking the soymilk at 100 °C for 20 min. Cold soaking of the soybeans was performed to inhibit microbial growth, so that the soaked water could be used directly in the grinding step to help recover leached isoflavones and other phenolics in the soaked water, specifically for grinding methods 3 and 4 listed below.

### 2.3. Specific Processing (Soaking and Grinding) Methods

Four different combinations of soaking and grinding methods were designed to produce soymilk and okara from soybeans, with the objective of recovering more phenolics from soymilk [26]. The soaking, grinding, or re-grinding details for the four methods (Supplementary Materials, Table S1) were presented in our previous publication [26]. To facilitate an understanding by readers and for ease of discussion, these (soaking and grinding) methods are referred to as ‘methods 1 to 4’ throughout this manuscript. For each method, the total water-to-dry soybean ratio was 10:1 (*w*/*w*). Briefly, the four methods were as follows.

Method 1: Tap water was used to grind soaked soybeans after draining (the traditional method served as the processing control).

Method 2: Okara-washed water was used to grind the soaked soybeans.

Method 3: Soaked water was used to grind the soaked soybeans (water-to-bean ratio of 10:1, *w*/*w*).

Method 4: Soaked water was used for the first grinding at a 6:1 (*w*/*w*) water-to-bean ratio and then filtered, then the okara was re-ground with tap water at a 4:1 (*w*/*w*) water-to-bean ratio.

All soymilk manufacturing methods were carried out at three different times and dates (replicates). A small portion of each raw soymilk sample was frozen and freeze-dried. After cooling the cooked soymilk to room temperature (around 20 °C), the cooked soymilk and okara were immediately frozen and freeze-dried. The soymilk yields and recovery rates of solids and proteins from the soybeans were calculated and expressed on the basis of 100 g of dry soybean used for processing.

### 2.4. Solvent Extraction of Phenolic Substances

The method published by Xu and Chang [27] for extraction from soy materials was used. Freeze-dried soymilk powder or okara was extracted in a 50:50 (*v*/*v*) acetone/water mixture for 3 h. The mixture was centrifuged and the supernatant was then saved. The residues were re-extracted and centrifuged to obtain the supernatants. Both supernatants were then combined and a portion was directly used for the total phenolic substance analyses. The solvent in the rest of the extract was removed via rotary evaporation at 38 °C under vacuum conditions. The concentrate was lyophilized to obtain a powder (referred to as crude phenolic extract hereafter) and stored in dark containers at −20 °C until performing further analyses of the individual phenolic acids and isoflavones using HPLC and anti-proliferation assays using cell cultures.

### 2.5. Determination of Total Phenolic Content (TPC) Values

The total phenolic content in each phenolic extract was determined using the Folin–Ciocalteu assay as described by Xu and Chang [27]. The TPC is expressed here as milligrams of gallic acid equivalents per gram of dry sample (mg of GAE/g).

### 2.6. Determination of Total Flavonoid Content (TFC) Values

The total flavonoid content was determined using a slightly modified colorimetric method described previously [32]. All values are expressed as milligrams of catechin equivalents per gram of dry sample (mg of CAE/g).

### 2.7. Determination of Condensed Tannin Content (CTC) Values

The condensed tannin content was determined according to the method reported by Xu and Chang [27]. The absorption was measured at 500 nm against methanol as a blank. All values are expressed as milligrams of catechin equivalents per gram of dry sample (mg of CAE/g).

### 2.8. Quantification of Major Soy Phenolic Acids

The freeze-dried crude phenolic extract was diluted in water to a concentration of 20 mg/mL for an analysis of phenolic acids using an Agilent 1200 Series HPLC system (Agilent Technologies, Santa Clara, CA, USA), using a Zorbax Stablebond Analytical SB-C18 column (250 × 4.6 mm, 5 μm, Agilent Technologies, Santa Clara, CA, USA) at 40 °C. The elution was performed using mobile phase A (0.1% trifluoroacetic acid aqueous solution) and mobile phase B (100% methanol) at a flow rate of 0.7 mL/min. The spectra were analyzed from 220 to 600 nm using a DAD at 270 nm. The elution gradient used here was 5–30% B over 50 min. The elution gradient was then held at 30% B for 15 min, increased to 100% B within 66 min, and then held at 100% B for 10 min for column cleaning, followed by column re-equilibration for 5 min with 95% A and 5% B before the next run. Authentic standards were used for the identification of phenolic compounds. The phenolic acid contents are expressed as micrograms of phenolic acid per gram of soymilk (μg/g) on a dry basis.

### 2.9. Quantification of Isoflavones using a High-Performance Liquid Chromatography (HPLC) Analysis

The isoflavones in the dried soymilk and okara samples were analyzed using an internal standard calibration method, as per the publications by Hou and Chang [33] and Xu and Chang [28]. Authentic standards were used for the identification of isoflavones.

### 2.10. Chemical Antioxidant Assays

The oxygen radical absorbance capacity (ORAC)*,* ferric-reducing antioxidant power (FRAP), and radical DPPH scavenging activity (DPPH) profiles were assayed to study the quantitative effects of processing on the antioxidant capabilities of soymilk. The ORAC was determined using the method used by Xu and Chang [27] and the results are expressed as micromoles of Trolox equivalents per gram of dry sample (μmol of TE/g). The FRAP assay was carried out following the method used by Benzie and Strain [34] (1996) and the results are expressed as millimoles of Fe^2+^ equivalents (FE) per 100 g of dry sample. The DPPH radical scavenging activity was measured following the method used by Xu and Chang [27] and the results are expressed as micromoles of Trolox equivalents per gram of dry sample (µmol of TE/g).

### 2.11. Anti-Proliferation Assays

The DU145 human prostate cancer cell line was used to study the biological activity of the phenolic extract, which contained isoflavones and other phenolic compounds, using the MTT assay as described by Tan et al. [26]. The absorbance of the cell suspension was measured at 570 nm using a Bio-Tech microplate reader. The cell viability was obtained by determining the difference in the absorbance values between treated and control wells divided by the absorbance value of the control.

### 2.12. Statistical Analysis

All processing methods and analyses were conducted in triplicate. The data are expressed as means ± standard deviations. The data were subject to a one-way analysis of variance (ANOVA). When significant differences among groups were detected, a post hoc Tukey’s test was used when more than two groups were compared at *p* < 0.05. The factors included the processing methods (1 to 4) and processed products (raw soymilk, cooked soymilk, and okara). When correlations between factors were needed, the Pearson’s correlation coefficients were analyzed. The significance level for all tests was set at *p* < 0.05. Various software packages (e.g., SigmaStat v.3.5, Sigmaplot v. 15.x, and SAS Software v.9.4) were used to perform the statistical analyses and to assess the significance of the data.

## 3. Results and Discussion

### 3.1. Yields of Soymilk and Recovery Rates of Solids and Proteins from Soybeans

**Figure 1:**
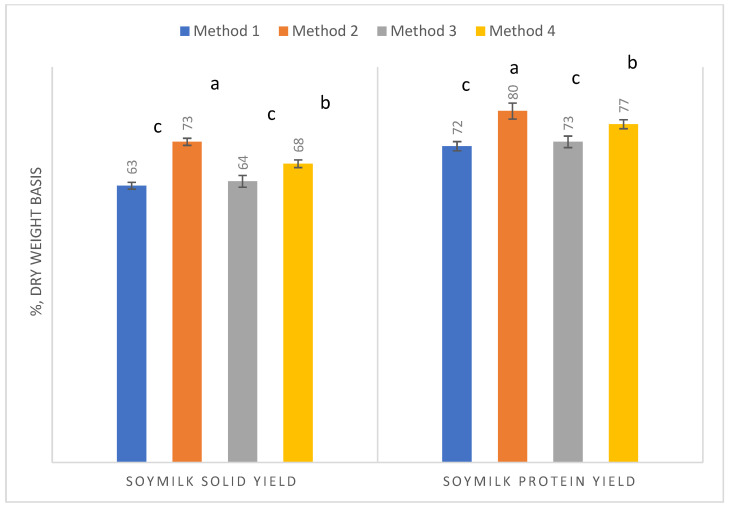
Soymilk solid and protein yields as affected by the four processing methods. Error bars represent standard deviations, while different lower-case letters within each group of soymilk solid and protein yields indicate significance at *p* < 0.05.

The results showed the (as is) soymilk recovery rates (processing yields) from 100 g of soybean raw materials were 968, 979, 968, and 992 g for methods 1, 2, 3, and 4, respectively. Method 4, a two-stage grinding method, involving first grinding soaked soybeans with soaked water (600 mL) and then re-grinding the okara with tap water (400 mL), gave the highest yield (*p* < 0.05) of soymilk (992 g) among the four manufacturing technologies. The second highest soymilk yield (significantly higher than methods 1 and 4; *p* < 0.05) was for method 2, which gave 979 g by using okara-washed water to grind soaked soybeans. Unlike the soymilk processing yields, the % yields of solids and protein were the highest using method 2 (Figure 1). Method 2 gave about 72.6% solids versus 62.8% for method 1 (traditional method using tap water grinding of the soaked beans) and gave 80.4% protein recovery versus 72.1% for method 1. These results represent 15.6% and 11.6% increases of the solid and protein recovery rates from the soybean mass over the traditional method (method 1). This study was the first quantitative characterization of increases using a single-step okara re-washing process to improve the solid and protein yields for yellow soybean. The ProSoy variety also gave significantly higher soymilk yields and higher solid (increased by 7.5%) and protein (increased by 7.2%) recovery rates when prepared using method 4. However, method 2 consistently gave the highest solid and protein recovery rates for both the ProSoy (yellow) soybean and black soybean [26].

### 3.2. Phenolic Processing Yields and Compositions

#### 3.2.1. Total Phenolic Content (TPC) Values

The TPC results for the raw soymilk, cooked soymilk, and okara from ProSoy soybeans using the four soymilk grinding methods are shown in Table 1. The TPC value for the raw soymilk made using method 1 (control), the traditional grinding method with tap water, was 1.94 mg GAE/g dried soymilk. The other three grinding methods produced soymilks with higher TPC values, particularly methods 3 and 4, with significant differences (*p* < 0.05). Among the methods, method 4, involving two-step grinding, gave the highest TPC value (*p* < 0.05) for raw soymilk at 2.61 mg GAE/g, which represented a 35% increase over the control method.

Cooking at 100 °C for 20 min reduced the TPC values by about 13–17% across the four methods. For the cooked soymilks, the TPC value of the soymilk ground using method 4 was significantly (*p* < 0.05) higher than those produced using methods 1 and 2. No significant differences were observed among the okara samples.

Table 2 shows the % recovery rates and losses of total phenolics from the ProSoy soybeans during soymilk processing. When the data were calculated based on the percentage of soybean TPC that was retained in dried soymilk, greater recovery rates (*p* < 0.05) of total phenolics were observed in raw soymilk (ranging from 43 to 63%) than cooked soymilk (ranging from 37 to 52%). Method 4 preserved much more of the TPC in the raw (increased by 44.5% over control; *p* < 0.05) and cooked (increased by 40.5% over method 1; *p* < 0.05) soymilks than the other three methods.

#### 3.2.2. Total Flavonoid Content (TFC) Values

The TFC values for the ProSoy soymilks are presented in Table 3. For the raw ProSoy soymilks, grinding method 4 caused a significantly (*p* < 0.05) greater TFC value than the other three grinding methods. However, no significant differences were observed among raw soymilks produced using grinding methods 1, 2, and 3. For the cooked soymilks, no statistically significant differences were observed among the soymilks produced using the four grinding methods, even though methods 4 and 2 produced higher values. Considering that methods 4 and 2 had higher processing, solid, and protein yields, the overall contribution to consumers were still more positive than the other two methods. The TFC value of the okara produced using grinding method 4 was higher (*p* < 0.05) than for the other three grinding methods, indicating that some flavonoids were preferentially retained in method 4’s okara. Cooking increased the TFC values from the range of 0.25–0.37 mg of CAE/g to 0.38–0.43 mg of CAE/g, an average increase of approximately 31%.

#### 3.2.3. Condensed Tannin Content (CTC) Determination

The CTC values for the ProSoy soymilks are presented in Table 4. For the raw soymilk, method 4 produced the highest (*p* < 0.05) CTC value of all grinding methods. The CTC values for the soymilks manufactured using methods 2 and 3 were greater (*p* < 0.05) than that of Method 1. The CTC values for the cooked soymilks processed using grinding methods 2 and 4 were greater (*p* < 0.05) than those of grinding methods 1 and 3. The CTC value of the okara produced using method 4 was greatest (*p* < 0.05) among the four grinding methods. The okara produced using method 3 had a greater (*p* < 0.05) CTC value than those of methods 1 and 2. We did not know why method 4 produced okara with higher CTC and TFC values than with the other three methods. A possibility was that the different grinding methods might have influenced the solvent extraction of these phenolic compounds from okara during the analyses.

#### 3.2.4. Free Phenolic Acid Compositions

The phenolic acid contents of the soymilks produced from ProSoy soybeans are presented in Table 5. Five phenolic acid components were detected in the ProSoy soymilks over a range of 84–106 μg/g soymilk (dry basis). Among the phenolic acids detected, gallic acid (GA), chlorogenic acid (CLA), and vanillic acid (VA) were the major ones. Method 4 and method 3 retained more phenolic acids than the other three grinding methods. Similar to the above-described phenolic substances, cooking had a negative effect on the retention of phenolic acids and the damage level was high, destroying approximately 50% of the phenolic acids in the soymilks (*p* < 0.05).

### 3.3. Isoflavone Compositions

The isoflavone contents of the ProSoy soymilk and okara samples produced using the four different processing methods are presented in Table 6. In general, with each grinding method, the cooked soymilk gave the highest isoflavone content among the raw, cooked, and okara samples. Cooking did not reduce but rather slightly increased the isoflavone content in the ProSoy soymilks, indicating that isoflavones are quite resistant to the traditional heating conditions of 100 °C for 20 min. Among the grinding methods, the results showed that method 4 and method 3 retained more isoflavones (*p* < 0.05) in the cooked soymilks than the other two grinding methods, with method 4 gave about 11.5% higher total isoflavones than the control and method 2.

It is well known that isoflavones may engage in interconversion between malonyl-glucoside and glucoside conjugate forms. Within each form of isoflavones, thermal conversion occurred, meaning that the malonyl-glucoside form was reduced with a concomitant increase in the glucoside conjugates. The aglycones did not change much after cooking at 100 °C for 20 min. The okara samples retained lower levels of isoflavones as compared to the soymilks; the okara made using method 4 had the lowest isoflavone. This was logical, since a greater amount of isoflavones was extracted into the soymilk using method 4.

### 3.4. Antioxidant Activity Profiles

The antioxidant activity results are presented in Table 7. Significant (*p* < 0.05) differences in the ORAC, FRAP, and DPPH values were found among most samples. For the raw soymilks, grinding methods 2, 3, and 4, significantly (*p* < 0.05) increased the ORAC, FRAP, and DPPH values compared to the traditional method (method 1). The soymilk produced using grinding method 4 especially exhibited higher (*p* < 0.05) antioxidant activity (FRAP and DPPH) values than the other three grinding methods. Cooking generally increased all of the tested antioxidant properties. For the cooked soymilks, grinding methods 3 and 4 produced significantly (*p* < 0.05) higher antioxidant activity levels (ORAC and FRAP values) than grinding methods 1 and 2. The raw soymilk produced using grinding method 4 exhibited the highest DPPH values among all soymilk samples, with a 150% higher value than that of method 1. The cooked soymilk made using method 4 had a 73% higher value than that of method 1. Another distinct advantage of method 4 was in the ORAC values, showing 47% higher antioxidant capacity than for method 1. Overall, the antioxidant properties were in the ranking order of method 4 ≥ method 3 > method 2 = method 1.

For the okara residues produced using all grinding methods, no significant differences were found in the antioxidant activity rates (ORAC and FRAP values); however, the DPPH value for grinding method 3 was significantly (*p* < 0.05) greater than that for the other three methods (Table 7).

### 3.5. Anti-Proliferative Properties of Soymilk against Human Prostate Cancer Cell Lines

The anti-proliferative properties of the ProSoy soymilks produced using the four processing methods against prostate cancer cells are summarized in Table 8. In this study, with a focus on the effect of grinding, among the tested ProSoy soymilk and okara samples, the raw soymilks also exhibited the highest anti-proliferative capacity rates as compared to the cooked soymilk and okara samples. In most cases, the okara samples exhibited the lowest anti-proliferative capacity rates among all samples, as indicated by the highest IC_50_ values. The raw ProSoy soymilk produced using grinding method 4 possessed the strongest (*p* < 0.05) anti-proliferative capacity against prostate cancer cells (4.9 mg/mL, lowest IC_50_) (Table 8), followed by the soymilks produced using grinding methods 2 and 3.

## 4. Discussion

### 4.1. Processing Yields and Solid and Protein Recovery Rates

Soymilk is a popular non-dairy beverage that has potential health benefits derived from its isoflavones and antioxidant phenolic substances. The health components and functions are affected by the soybean variety, and most notably by the soybean color, namely between yellow and black soybeans. The results from this work in some parts are the first confirmation of our previously published work [26] on black soymilks extracted using the four soaking and grinding methods. However, there were significant differences in processing yields and protein yields, in which the yellow ProSoy soybean had a clear advantage.

As stated in our previous publication on black soymilk [26], one of the major objectives of studying various methods of soaking, grinding, washing, and re-grinding okara was to recover better yields and more health-promoting materials from the soybean. We were able to accomplish these goals with black soymilk. However, the effect and significance of the product yields were not fully discussed in our previous report [26]. Here, we provide a full analysis of the higher yields from these different soymilk manufacturing methods.

When we compared the results to the soymilk yields from black soybean, we found that the yields for the ProSoy soymilks were consistently higher than with the respective methods for black soybean extraction [26], which yielded values of 949, 958, 974, and 956 g/100 g bean, respectively. ProSoy gave higher yields, which may have been partically due to greater water absorption during soaking at 4 °C before grinding, since the ratios of soaked weight/raw dry weight were about 2.28 versus 2.15 for black soybean. In our previous research, we found that the soaked weight was related to the total soluble solid and protein contents that can be extracted into aqueous soymilk systems [35].

As compared to method 2 for black soymilk [26], the ProSoy soymilk made using method 2 gave higher solid and protein recovery rates in the soymilk, since the solid and protein recovery rates for method 2 in black soymilk were approximately 10% each. This study is the first quantitative characterization of increases from using a single step of okara re-washing to improve the solid and protein yields for yellow soybean.

However, the most striking differences between the ProSoy and black soybeans were with method 4, whereby the black soybean gave much lower soymilk solid and protein recovery rates (61.5% and 70.1%, respectively, for black soybean, as compared to 67.6 and 77.3% for ProSoya). These respectively represented 9.92% and 10.3% higher solid and protein yields over black soymilk, which are very important results economically, since smaller ProSoy yellow soybean amounts (approximately lower 10% dry bean weight) can achieve the same soymilk concentrations, thereby benefiting yellow soybean manufacturers by comparatively lowering the raw material costs. In addition to the decreased raw material costs, the solid yield results are particularly important, since the antioxidant and health-promoting activities reported in the following sections are based on the per gram yield of soymilk solids. Therefore, when a processing technology gives higher solid yields in soymilk, it will provide more beneficial effects to the soymilk manufacturer and consumers. Since yellow soybeans have higher planting yields than black soybeans, the net economic impact to the farmers is greater.

### 4.2. Phenolic Processing Yields and Compositions

The components that have been widely reported in soybean materials consist of the total phenolic content, flavonoids, phenolic acids, and condensed tannins, which were reported in many soybean varieties and sources in our previous study [9]. Anthocyanins do not exist in yellow soybean in significantly volumes. Most studies have reported on the contents using materials studies, while few have reported on the recovery rates of phenolic compounds as affected by the soymilk manufacturing method.

The TPC for the raw ProSoy soymilk was lower than the TPC values of 2.34 and 2.79 mg GAE/g, respectively, for the raw soymilks from the Proto and IA 2032 yellow soybean cultivars, which were made using the same processing method as described in our previous study [28]. The cooking effect on the TPC values was consistent with the percentage reductions for the cooked soymilks made from the Proto and IA2032 yellow soybeans [28]. We previously reported substantially greater losses (23–38%) when cooking soymilks made from black soybean while using the same four grinding methods used in this study [26]. This clearly showed the characteristic differences between yellow and black soybeans.

The overall trend for the processing effect was similar to that observed in black soybean soymilk manufacturing [26]. However, the total TPC content in the ProSoy soymilk was lower than that in the black soybean soymilk, which is known to contain anthocyanins [31], whereas yellow soybeans do not have anthocyanins. It is well known that anthocyanins are sensitive to heat [36] and can be easily destroyed, particularly at near-neutral pH levels (in soymilk this is a pH of around 6.5), which may have been partially responsible for greater losses of black soymilk from cooking.

The results from the phenolic recovery studies suggested that the double grinding method facilitated the greater release of phenolics from the soybean matrix. These findings are important, since method 4 gave increased yields of soymilk products, including the solid (8%) and protein (7%) contents discussed above. Method 2 also seemed to have a higher TPC recovery tendency than the control soymilk (20% TPC increase over the cooked control soymilk). Therefore, together with the increases in production, solid, and protein yields (15.6, 11.6, and 11.6% over the control (method 1), respectively), method 2 also produced significantly more benefits than the traditional method in the retention of phenolics from the raw soybean materials.

Cooking generally reduced the phenolic compounds. However, cooking increased the total flavonoid values by approximately 31%. The reasons for the increases may have been because some protein-bound flavonoids [37] were released, making them more extractable during the TFC determination process [9,28]. Compared to the TFC values for the cooked Proto soymilk (0.16–0.17 mg CAE/g) [28], the ProSoy soymilk had a much higher TFC.

Among the phenolic acids detected here, gallic acid (GA), chlorogenic acid (CLA), and vanillic acid (VA) were the major ones. These phenolic acids were detected in our previous soymilk research [28] and have been reported to be among the major ones in the soy research by Yu et al. [23]. When the ProSoy soybeans were made into soymilk, most of the free phenolic acids became undetectable. We do not know the reasons for this finding. Further studies are needed to understand this phenomenon. However, when we compared the values for the soymilks made using the four grinding methods, we found the method 4 and method 3 retained more phenolic acids than the other three grinding methods. As discovered for the previously discussed phenolic substances, cooking had a negative effect on the retention of phenolic acids, and the damage rate was high, destroying approximately 50% of the phenolic acids in the soymilks (*p* < 0.05).

### 4.3. Isoflavone Compositions

There are three basic structures (dadzein, genistein, and glycitein) and 12 individual forms of isoflavones in soybeans, since each structure exists in aglycone, glucoside, acetyl glucoside, and malonyl-glucoside forms. We did not analyze the acetyl-glucoside isoflavones, since they are very minor as compared to the malonyl-glucoside, glucoside, and aglycone forms [9,28].

The isoflavone results in general are consistent with the increasing patterns for the TPC, TFC, and CTC values using method 4. This was also observed in the previous study by Tan et al. [26] with black soybeans. The ProSoy soymilks exhibited higher (*p* < 0.05) total isoflavone contents than the black soymilks, as reported in our previous study [26]. This might have significance for health promotion, since isoflavones, particularly genistein and dadzein, have been well known to be related to the prevention of chronic diseases. Li and Sharkar [38] studied the effect of purified genistein and reported 70% inhibition of PC3 cell growth at 50 μmol/L (equivalent to 13.5 μg/mL). A much higher concentration of genistein was reported by Yu and others [39] as being able to completely inhibit the expression of prostate androgen-regulated transcript-1 at 50 mmol/L (13.5 mg/mL). The large literature discrepancies regarding the effectiveness of isoflavones against cancer cell proliferation may be due to the different cell lines and sources of materials. Our testing materials were crude phenolic extracts, which contained mixtures of different types of polyphenolic compounds. Hsu and others [22] reported that some synergistic effect of various types of isoflavone compounds may exist in a whole soy extract in the inhibition of the DU145 prostate cancer cell line.

### 4.4. Antioxidant Activity Profile

Oxidative stress has long been regarded as a major contributor to cancer generation, as oxidants have the ability to induce DNA damage and stimulate cell division. Antioxidants help protect cells from uncontrolled cell division. Soy phenolics act as natural antioxidants to promote health. Three assay methods (ORAC, FRAP, and DPPH) with different antioxidative reaction mechanisms (based on single-electron transfer and hydrogen atom transfer) were used to determine the profiles of the antioxidant properties of the soymilk and okara samples made using the four grinding methods. It is well known that analyzing antioxidant patterns with multiple chemical assays with different reaction mechanisms better reflects the potential of the phenolics’ biological functions [40].

The antioxidant activity patterns for the ProSoy soymilks were consistent with our earlier findings [26] for black soymilks. It had been reported that thermal treatments could induce the formation of compounds with new antioxidant properties [41]. An increase in antioxidant activity from thermal treatment was also found in the pasteurization of tea extracts [42].

### 4.5. Anti-DU145 Prostate Cancer Cell Proliferation

In this study, we focused on the effect of grinding among ProSoy soymilk and okara products, whereby raw soymilks exhibited higher anti-proliferative capacity rates than cooked soymilk and okara samples. In most cases, the okara samples exhibited the lowest anti-proliferative capacity among all samples, as indicated by having the highest IC_50_ values. The raw ProSoy soymilk produced using grinding method 4 possessed the strongest (*p* < 0.05) anti-proliferative capacity against prostate cancer cells (4.9 mg/mL, lowest IC_50_) (Table 8), followed by the soymilk produced using grinding methods 2 and 3. Unfortunately, raw soymilk cannot be consumed, since it contains anti-nutrients such as trypsin inhibitors and lectins [24]. Raw soymilk also must be cooked to improve the flavor by destroying beany-odor-producing lipoxygenases. The results showed that cooking at 100 °C for 20 min decreased the anti-prostate-cancer potential of the soymilks made using methods 3 and 4. However, the IC_50_ value of the cooked soymilk made using grinding method 4 was the lowest, even though no statistical differences were seen with methods 1 and 2. Cooking at 100 °C for 20 min did not seem to affect the potential of the soymilks made using methods 1 and 2. We do not know exactly why the soymilks made using different grinding methods responded differently to cooking, and presumably the extracted components differed. This also seems to hold true for black soymilk [26]. Furthermore, in our previous study, we found that an industrial process, namely a two-stage heating method consisting of 120 °C for 80 s + 140 °C for 4 s, did not reduce the potency of the anti-DU145 cell proliferation as compared to raw soymilk [43]. Presumably, the differential responses to various grinding and thermal processing methods might be related to the heat sensitivity of the antioxidants or other extractable materials that contribute to the overall anti-cancer effect. The phenomenon deserves further research in the future. The IC_50_ values for all okara samples produced using the four grinding methods were not significantly different. This is consistent with the values reported for okara in black soymilk processing [26].

Like those of the black soymilk extracts, the crude phenolic extracts from all ProSoy samples exhibited the ability to inhibit the proliferation of DU 145 prostate cancer cells in vitro, although the black soymilks were more effective, with IC_50_ values of 4–7 mg/mL as compared to the ProSoy IC_50_ values of 6.8–10.1 mg/mL (cooked soymilks). Interestingly, there was a similar anti-proliferation pattern for method 3, which produced soymilks with the lowest anti-proliferation capacity rates from both black and ProSoy soybeans, while method 4 produced the lowest values (higher potency). Earlier, we reported that raw and processed soymilks made from the Proto variety inhibited the growth of DU145 cancer cells in a dose-dependent manner, while IC_50_ values were reduced by thermal processing and an industrial two-step heating method gave the lowest IC_50_ value [43]. Thermal processing in general lowers the inhibitive capability of soymilk.

## 5. Conclusions

This study is the first report to quantitatively characterize soymilks produced from food-grade yellow ProSoy soybeans, including the soymilk production, solid, and protein yields, in conjunction with the phenolic components, antioxidant capacity rats, and in vitro anti-prostate cancer cell properties, as affected by four methods of soymilk processing. The results showed significant characteristic differences from our previous research on black soymilk, which could produce a positive impact on soybean growers, the soymilk food industry, and consumers. Overall, the ProSoy soymilks showed distinct differences in yields. Method 2 and method 4 gave higher yields, which can be used by the soymilk industry to reduce the raw material costs. However, method 4 consistently gave better phenolic profiles and antioxidant capacity rates, which may benefit consumer health. When considering both the yields and health benefits, method 4 would be the first choice, since consumers can obtain more antioxidant phenolics and proteins in the same amount of soymilk. However, method 2 can also partly achieve the same goals as the traditional soymilk processing method, since the solid and protein yields were significantly higher. The yellow ProSoy soymilks made using the processing methods described in this study had higher contents of isoflavones than the black soymilks and good potential to inhibit DU145 prostate cancer cell proliferation. Future research should be conducted using animal or human clinical tests to further characterize the health functions of consuming soymilk or the use of dried crude phenolic extracts as dietary supplements for health improvement. Black soymilk should be used for comparison. If yellow soymilk could achieve similar health benefits to black soymilk, there would be less incentive to consume more expensive black soymilk.

## Figures and Tables

**Table 1:** Total phenolic content (TPC) values for ProSoy soymilk and okara as affected by the four soymilk manufacturing methods and cooking ^1^.

TPC Values (mg of GAE/g)			
Grinding	Raw Soymilk	Cooked Soymilk	Okara
Method 1	1.94 ± 0.04 cA	1.68 ± 0.14 bB	1.09 ± 0.17 aC
Method 2	2.09 ± 0.03 bcA	1.74 ± 0.04 bB	0.97 ± 0.03 aC
Method 3	2.11 ± 0.04 bA	1.84 ± 0.09 abA	1.02 ± 0.22 aC
Method 4	2.61 ± 0.11 aA	2.17 ± 0.25 aB	1.30 ± 0.03 aC

^1^ Data were calculated on a dry weight basis and are expressed as means ± standard deviations (n = 3). Values marked by different lowercase letters within each column are significantly different (*p* < 0.05). Values marked by the same uppercase letter within the same row are not significantly different (*p* < 0.05).

**Table 2:** Recovery of TPC values (% of that in soybean) in raw and cooked ProSoy soymilks and okara as affected by the four soymilk manufacturing methods ^1^.

Grinding	Raw	Cooked	Okara	Total ^2^	Loss ^3^
Method 1	43.47 ± 1.32 cA	37.14 ± 3.39 bB	12.49 ± 1.67 aC	55.95 ± 2.86 b	44.05 ± 2.86 b
Method 2	54.03 ± 1.20 bA	44.45 ± 1.21 abB	10.90 ± 0.67 aC	64.94 ± 1.62 ab	35.06 ± 1.62 ab
Method 3	48.06 ± 1.92 cA	41.63 ± 1.16 bB	11.45 ± 2.46 aC	59.51 ± 3.15 ab	40.49 ± 3.15 ab
Method 4	62.90 ± 2.81 aA	52.19 ± 6.86 aB	12.47 ± 0.48 aC	75.37 ± 2.56 a	24.63 ± 2.56 a

^1^ Data were calculated on a dry weight basis and are expressed as means ± standard deviations (n = 3). Values marked by different lowercase letters within each column are significantly different (*p* < 0.05). Values marked by different uppercase letters within the same row are significantly different (*p* < 0.05). ^2^ The “total” column represents the phenolic content from the raw soymilk and okara combined. ^3^ The “loss” column represents the difference in the total phenolic contents between the raw soymilk and the ProSoy soybean powder.

**Table 3:** TFC values for raw and cooked ProSoy soymilks and okara as affected by the four soymilk manufacturing methods ^1^.

TFC Values (mg of CAE/g)			
Grinding	Raw	Cooked	Okara
Method 1	0.25 ± 0.03 bC	0.38 ± 0.03 aB	0.63 ± 0.06 bA
Method 2	0.35 ± 0.03 bB	0.42 ± 0.04 aB	0.65 ± 0.10 bA
Method 3	0.26 ± 0.05 bB	0.38 ± 0.03 aB	0.76 ± 0.10 bA
Method 4	0.37 ± 0.04 aB	0.43 ± 0.03 aB	0.89 ± 0.05 aA

^1^ Data were calculated on a dry weight basis and are expressed as means ± standard deviations (n = 3). Values marked by different lowercase letters within each column are significantly different (*p* < 0.05). Values marked by the same uppercase letter within the same row are not significantly different (*p* < 0.05).

**Table 4:** Condensed tannin content (CTC) values for raw and cooked ProSoy soymilks and okara as affected by the four soymilk manufacturing methods ^1^.

CTC Values (mg of CE/g)			
Grinding	Raw	Cooked	Okara
Method 1	0.21 ± 0.02 dBA	0.14 ± 0.02 bB	0.46 ± 0.05 cA
Method 2	0.37 ± 0.05 bC	0.27 ± 0.03 aB	0.49 ± 0.01 cA
Method 3	0.29 ± 0.05 cC	0.13 ± 0.01 bB	0.60 ± 0.03 bA
Method 4	0.48 ± 0.02 aC	0.32 ± 0.03 aB	0.70 ± 0.07 aA

^1^ Data were calculated on a dry weight basis and are expressed as means ± standard deviations (n = 3). Values marked by different lowercase letters within each column are significantly different (*p* < 0.05). Values marked by the same uppercase letter within the same row are not significantly different (*p* < 0.05).

**Table 5:** Phenolic acid content (μg/g) values for raw and cooked ProSoy soymilks and okara as affected by the four soymilk manufacturing methods ^1^.

	GA	VA	CA	CLA	*p*-HBA	Total ^2^
**Raw**						
M1	29.5 ± 1.4 b	17.3 ± 1.6 ab	1.5 ± 0.1 a	35.9 ± 4.0 a	nd	84.2 ± 7.0 b
M2	31.4 ± 1.5 b	15.7 ± 1.1 ab	nd	37.4 ± 3.4 a	nd	84.5 ± 6.0 b
M3	37.1 ± 1.2 a	20.2 ± 1.3 a	1.3 ± 0.1 a	42.9 ± 5.5 a	nd	101.4 ± 8.2 a
M4	41.1 ± 0.9 a	14.0 ± 1.2 b	1.4 ± 0.2 a	48.5 ± 4.3 a	1.5 ± 0.2	106.5 ± 6.8 a
**Cooked**						
M1	22.0 ± 2.0 b	12.7 ± 0.6 b	0.9 ± 0.1 c	nd	nd	35.7 ± 2.7 b
M2	23.3 ± 1.1 b	12.3 ± 1.3 b	nd	nd	nd	35.6 ± 2.3 b
M3	29.4 ± 1.5 a	21.8 ± 1.3 a	2.5 ± 0.1 a	nd	2.1 ± 0.1	55.8 ± 3.0 a
M4	30.4 ± 1.1 a	24.6 ± 1.3 a	1.4 ± 0.1 b	nd	nd	56.4 ± 2.5 a
**Okara**						
M1	18.0 ± 0.9 a	nd	nd	nd	nd	18.0 ± 0.9 a
M2	17.2 ± 1.3 a	nd	nd	nd	nd	17.2 ± 1.3 a
M3	16.9 ± 1.3 a	nd	nd	nd	nd	16.9 ± 1.3 a
M4	13.5 ± 1.4 b	nd	nd	nd	nd	13.5 ± 1.4 b

^1^ Data were calculated on a dry weight basis and are expressed as means ± standard deviations (n = 3). The phenolic acids analyzed using HPLC included gallic acid (GA), vanillic acid (VA), caffeic acid (CA), chlorogenic acid (CLA), and *p*-hydroxybenzoic acid (*p*-HBA). Note: nd, not detectable. ^2^ “Total” represents the sum of the five phenolic acids listed in this table ± the standard deviation. Values marked by different lowercase letters across the four methods are significantly different (*p* < 0.05).

**Table 6:** Isoflavone content (μg/g) values for raw and cooked ProSoy soymilks and okara as affected by the four soymilk manufacturing methods ^1^.

	Din	Gly	Gin	MDin	MGly	MGin	MGly	Dein	Gein	Total ^2^
**Raw**										
M1	274.7 ± 2.3 c	43.9 ± 4.1 a	314.7 ± 17.1 b	625.4 ± 5.1 d	113.5 ± 7.7 a	1743.4 ± 49.5 a	119.5 ± 2.2 b	49.7 ± 4.9 a	62.0 ± 2.5 a	3346.8 ± 90.3 b
M2	260.5 ± 1.9 d	42.8 ± 1.1 a	317.0 ± 3.6 b	790.3 ± 52.8 c	110.7 ± 0.2 a	1705.9 ± 33.0 a	134.9 ± 6.7 b	53.6 ± 4.6 a	65.0 ± 3.3 a	3480.9 ± 34.1 b
M3	289.1 ± 3.8 b	48.3 ± 3.9 a	346.9 ± 6.9 ab	974.8 ± 3.7 ab	128.3 ± 1.2 a	1818.8 ± 16.5 a	130.3 ± 8.8 b	57.3 ± 0.6 a	63.7 ± 7.1 a	3857.6 ± 37.4 a
M4	333.1 ± 1.9 a	48.9 ± 0.5 a	372.3 ± 15.5 a	985.9 ± 22.0 a	134.5 ± 13.0 a	1812.5 ± 16.5 a	180.8 ± 5.3 a	62.9 ± 4.3 a	67.8 ± 5.9 a	3998.8 ± 84.9 a
**Cooked**										
M1	540.5 ± 5.7 b	69.6 ± 6.9 a	648.5 ± 12.1 b	653.3 ± 22.0 b	111.9 ± 5.0 a	1351.3 ± 18.1 ab	157.2 ± 4.8 a	39.9 ± 2.6 b	52.3 ± 2.2 a	3624.7 ± 65.4 b
M2	572.6 ± 24.5 ab	62.7 ± 6.9 a	636.5 ± 17.5 b	677.6 ± 22.4 ab	108.7 ± 2.5 a	1337.9 ± 19.8 b	162.0 ± 5.0 a	44.7 ± 4.3 ab	58.4 ± 3.3 a	3666.1 ± 42.7 b
M3	610.0 ± 20.0 ab	79.2 ± 1.4 a	672.0 ± 9.0 ab	715.9 ± 21.2 ab	120.8 ± 5.0 a	1384.2 ± 19.7 ab	173.3 ± 7.0 a	50.0 ± 3.9 ab	60.7 ± 2.2 a	3866.0 ± 29.5 a
M4	641.9 ± 12.6 a	78.1 ± 5.7 a	716.0 ± 3.1 a	754.5 ± 4.4 a	123.6 ± 2.5 a	1429.4 ± 28.0 a	183.9 ± 10.1 a	56.6 ± 2.9 a	58.5 ± 7.7 a	4042.5 ± 46.7 a
**Okara**										
M1	49.7 ± 0.6 b	22.1 ± 1.2 a	103.7 ± 5.9 a	353.1 ± 22.0 b	62.5 ± 2.5 a	749.8 ± 18.5 a	67.2 ± 6.6 a	124.3 ± 5.7 a	172.2 ± 12.0 a	1704.6 ± 71.9 a
M2	73.9 ± 3.7 a	24.6 ± 1.2 a	103.6 ± 3.1 a	352.8 ± 20.2 b	58.6 ± 3.5 a	661.4 ± 16.5 b	59.1 ± 5.3 a	106.9 ± 5.0 ab	152.3 ± 4.3 ab	1593.4 ± 12.1 a
M3	71.7 ± 3.8 a	25.1 ± 2.5 a	112.7 ± 5.8 a	338.1 ± 12.0 b	59.3 ± 1.5 a	714.3 ± 11.4 ab	62.6 ± 3.2 a	105.2 ± 1.4 ab	138.2 ± 7.6 b	1627.6 ± 14.9 a
M4	76.5 ± 3.8 a	22.0 ± 2.2 a	78.5 ± 6.6 b	239.1 ± 19.8	47.8 ± 0.7 b	477.3 ± 26.4 c	36.9 ± 5.4 b	101.5 ± 7.1 b	149.2 ± 4.3 ab	1229.1 ± 39.5 b

^1^ Data (microgram/g) were calculated based on dried crude phenolic extract isolated from soymilk and are expressed as means ± standard deviations (n = 3). Note: Din, daidzin; Gin, genistin; Gly, glycitin; MDin, malonyldaidzin; MGin, malonylgenistin; MGly, malonylglycitin; MGly, malonylglycitin; Dein, daidzein; Gein, genistein; nd, not detectable. ^2^ “Total” represents the mass (μg/g) sum of the nine different isoflavones detected using HPLC. Values marked by different lowercase letters across the four methods are significantly different (*p* < 0.05).

**Table 7:** Antioxidant profiles of raw and cooked soymilks and okara from ProSoy soybeans as affected by the four processing methods ^1^.

**ORAC Values (µmol TE/g)**			
**Grinding**	**Raw**	**Cooked**	**Okara**
Method 1	71.49 ± 4.26 bB	89.89 ± 1.68 cA	67.50 ± 6.13 aB
Method 2	75.36 ± 3.92 abC	101.80 ± 4.67 bcB	59.77 ± 2.14 aA
Method 3	88.25 ± 7.96 aC	109.65 ± 9.54 bB	56.40 ± 3.31 aA
Method 4	86.45 ± 6.20 abC	132.56 ± 4.26 aB	59.94 ± 5.00 aA
**FRAP Values (mmol Fe^2+^ Equivalents/100 g)**			
**Grinding**	**Raw**	**Cooked**	**Okara**
Method 1	0.92 ± 0.02 dC	1.08 ± 0.04 bB	0.78 ± 0.03 aA
Method 2	1.07 ± 0.04 cB	1.10 ± 0.03 bB	0.79 ± 0.02 aA
Method 3	1.01 ± 0.01 bC	1.25 ± 0.06 aB	0.77 ± 0.04 aA
Method 4	1.13 ± 0.04 aC	1.30 ± 0.09 aB	0.71 ± 0.03 aA
**DPPH Assay (µmol TE/g)**			
**Grinding**	**Raw**	**Cooked**	**Okara**
Method 1	0.58 ± 0.09 cB	1.13 ± 0.04 cA	0.43 ± 0.09 bB
Method 2	0.83 ± 0.03 bC	1.78 ± 0.07 baB	0.37 ± 0.04 bA
Method 3	0.77 ± 0.08 bB	1.57 ± 0.05 bA	0.73 ± 0.09 aB
Method 4	1.45 ± 0.12 aC	1.96 ± 0.21 aB	0.47 ± 0.07 bA

^1^ Data were calculated on a dry weight basis and are expressed as means ± standard deviations (n = 3). Values marked by the different lowercase letters within each column and within each antioxidant assay are significantly different (*p* < 0.05). Values marked by the same uppercase letter within the same row are not significantly different (*p* < 0.05).

**Table 8:** Anti-DU145 prostate cancer cell proliferation IC_50_ values (mg/mL) in the crude phenolic extracts from raw soymilk, cooked soymilk, and okara samples from ProSoy soybeans ^1^.

IC_50_ Values (mg/mL) of MTT Assay			
Grinding	Raw	Cooked	Okara
Method 1	8.5 ± 0.7 cA	8.1 ± 0.6 bA	9.6 ± 0.7 aA
Method 2	7.7 ± 0.7 bcA	7.7 ± 0.9 bA	8.4 ± 0.3 aA
Method 3	7.0 ± 0.6 bB	10.1 ± 0.6 aA	9.5 ± 1.2 aA
Method 4	4.9 ± 0.2 aB	6.8 ± 0.1 bA	9.4 ± 0.7 aA

^1^ Data were calculated on a dry weight basis and are expressed as means ± standard deviations (n = 3). Values marked by different lowercase letters within each column are significantly different (*p* < 0.05). Values marked by the same uppercase letter within the same row are not significantly different (*p* < 0.05).

## Data Availability

The data are not publicly available due to the institutional policies.

## Supplementary Materials

The following supporting information can be downloaded at https://www.mdpi.com/article/10.3390/antiox13070755/s1: Table S1. Detailed description of the four grinding methods used for making soymilk.
