# Supplementary material for: Mass Yields, Antioxidant and Anti-DU145 Prostate Cancer Cell Proliferation Properties of ProSoy Soymilk as Affected by Extraction Methods and Cooking
Source: Antioxidants (Basel). 2024 Jun 21;13(7):755. doi: 10.3390/antiox13070755 (PMC11273802; doi:10.3390/antiox13070755)
Supplement: Supplementary file 1 [file antioxidants-13-00755-s001.zip › antioxidants-3025918-supplementary.pdf]

Supplemental Materials

Table S1. Detailed description of four grinding methods for making soymilk.

Soaking/Grinding	Method 1	Method 2	Method 3	Method 4
Soaking[a] and Vol. of soaked water	600 mL, Free soaked water was discarded	600 mL, Free soaked water was discarded	1000 mL, Soaked water was kept for grinding	600 mL, Soaked water was kept for grinding
First grinding[b] (3 min)	With tap water so that water to bean ratio is 10:1 (w/w)	With 200 mL of tap water absorbed in the bean plus 800 mL of okara-washed water[c] so that final water to bean rario is 10:1 (w/w)	With 1000 mL of soaked water (water to bean ratio is 10:1, w/w)	With 600 mL of soaked water (water to bean ratio is 6:1, w/w in the first grinding)
Re-grinding (3 min)	No re-grinding	No re-grinding	No re-grinding	Regrinding after adding 400 mL of tap water to okara so that final water to bean ratio is 10:1 (w/w)
Weight of soybean	100 g for each replicate for all methods			

[a] Soaking for all Methods was carried out at 4 C. Soybean was thoroughly rinsed before soaking.
[b]All grinding operation was carried out at the ambient temperature.
[c]Okara, the residue after filtering the slurry from the previous run was washed with tap water gently for one minute to produce okara-washed water for grinding.
